# Auricular Vagus Nerve Stimulation Combined with Physical Therapy for Individuals with Parkinson’s Disease: A Pilot Randomized Sham-Controlled Trial

**DOI:** 10.3390/neurolint18060118

**Published:** 2026-06-17

**Authors:** Alexandra Evancho, Jennifer Dawson, Harrison C. Walker, Christopher G. Ballmann, William J. Tyler

**Affiliations:** 1 Department of Physical Therapy, University of Alabama at Birmingham, Birmingham, AL 35294, USA; cgb0002@uab.edu; 2 Center for Engagement in Disability Health and Rehabilitation Sciences, University of Alabama at Birmingham, Birmingham, AL 35294, USA; 3 Center for Neuroengineering and Brain Computer Interfaces, University of Alabama at Birmingham, Birmingham, AL 35294, USA; hcwalker@uabmc.edu (H.C.W.); wjpt@uab.edu (W.J.T.); 4 Birmingham Veterans Affairs, Birmingham, AL 35294, USA; 5 Department of Neurology, University of Alabama at Birmingham, Birmingham, AL 35294, USA; 6 Department of Human Studies, University of Alabama at Birmingham, Birmingham, AL 35294, USA; 7 Department of Biomedical Engineering, University of Alabama at Birmingham, Birmingham, AL 35294, USA

**Keywords:** Parkinson’s disease, physical therapy, taVNS

## Abstract

**Background:** Both neuromodulation and physical therapy have been shown to mitigate motor and non-motor symptoms of Parkinson’s disease. To date, no studies have examined the integration of transcutaneous auricular vagus nerve stimulation (taVNS) with physical therapy approaches for improving Parkinsonian symptoms. The purpose of this study was to investigate the safety, tolerability, and feasibility of combining taVNS with physical therapy to enhance the therapeutic benefits of exercise as medicine in a clinical setting. **Methods:** Participants were randomly assigned to receive active or sham bilateral taVNS in combination with PT for 12 visits over 6 weeks. Safety, tolerability, and feasibility outcomes were primary. Secondly, exploratory analyses of changes in cardiovascular and motor function over time were also performed. **Results:** Overall, taVNS was safe and well-tolerated prior to PT. Cardiovascular analyses suggest that active taVNS may augment HR response to exercise compared to sham. For motor outcomes, both groups showed significant overall improvements; however, no significant between-group differences were found. **Conclusions:** The preliminary results obtained in this pilot trial confirm that taVNS combined with physical therapy for individuals with PD is safe and feasible. The exploratory cardiovascular and motor findings support the need for larger, adequately powered clinical trials investigating the integration of taVNS into PT and exercise methods for improving PD symptomology. Trial registration: ClinicalTrials.gov NCT05871151.

## 1. Introduction

Parkinson’s disease (PD) is a prevalent, progressive neurodegenerative disease classically characterized by degeneration of nigrostriatal dopaminergic neurons [1]. Although PD has historically been categorized as a movement disorder, it is now recognized as a multisystem disease with highly prevalent non-motor symptoms, including dysautonomia, sleep disturbance, anxiety, and depression [2,3,4]. These symptoms reflect the widespread nature of PD pathology, which extends beyond the nigrostriatal dopaminergic system and involves broader autonomic, inflammatory, and metabolic processes that may contribute to disease progression and symptom burden [5,6,7]. Although dopamine replacement therapy remains a first-line treatment and provides meaningful benefit for many motor symptoms, it does not fully address the multisystem manifestations of PD. 

While not curative, exercise is a well-established non-pharmacologic therapy that can improve cardiovascular and metabolic health [8], reduce systemic inflammation [9], and enhance neuroplasticity [10,11], effectively targeting both motor and non-motor symptoms [12,13]. Physical Therapy (PT) is frequently prescribed for PD patients and provides a structured, reimbursable setting for exercise. However, implementing evidence-based exercise protocols into routine PT care is often challenging [14,15]. For example, LSVT BIG therapy, which is a well-established evidence-based exercise protocol specifically designed for PD patients [16,17,18], requires 16 h-long, in-person visits over the course of 4 weeks. If the patient who has been prescribed this treatment lives in a remote area, lacks consistent caregiver support, and/or works full-time, this treatment frequency will not be achievable. Additionally, high-intensity aerobic exercise, which is recommended in published clinical practice guidelines for PT management of the PD patient [19], can also be challenging. To achieve neuroplastic benefit, the patient must reach and sustain moderate-to-high training intensities [20,21]; however, non-motor symptoms such as apathy, fatigue [22] and cardiovascular dysautonomia [23,24,25] may limit their ability to do so. In both scenarios, environmental, social, and/or physiologic barriers interfere with the patient’s ability to achieve the full therapeutic dose of exercise required for meaningful effect. These barriers contribute to a wide gap between improvements observed in clinical research and outcomes achieved in routine clinical practice, raising an important question: can we increase the therapeutic impact of PT treatment so that the benefits of exercise are more accessible in a clinical setting?

Neuromodulation is a promising therapeutic approach that is increasingly being explored as an add-on to rehabilitation to enhance recovery and restore function for patients with neurological conditions [26]. In particular, pairing vagus nerve stimulation (VNS) with rehabilitation has been shown to augment functional recovery, indicated by greater improvements with rehab + VNS compared to rehabilitation alone [26,27,28,29,30,31,32,33,34]. VNS traditionally involves the delivery of electrical currents to the cervical portion of the vagus nerve (VN) in the neck via a pulse generator that is surgically implanted in the chest wall. While promising, the reach of this approach is limited by surgical risks and associated costs. Alternatively, transcutaneous auricular vagus nerve stimulation (taVNS) is a non-invasive alternative that targets the auricular branch of the vagus nerve (ABVN) through the skin of the external ear [35,36,37]. Given physical therapists’ familiarity with transcutaneous electrical stimulation therapies, taVNS is a more practical, lower-risk, and potentially scalable adjunct to rehabilitation interventions for patients with neurologic diagnoses, including PD.

Stimulation of the ABVN is thought to engage vagal afferent pathways that project centrally to the nucleus tractus solitarius (NTS) [38], a primary brainstem relay for vagal sensory input. The NTS has broad connections with forebrain, limbic, and brainstem regions, including projections to the locus coeruleus (LC), the brain’s major noradrenergic nucleus and a putative mediator of VNS-related therapeutic effects. Through this pathway, taVNS may modulate LC–norepinephrine signaling, which is implicated in arousal, attention, and neuroplasticity [39], providing a plausible mechanism by which taVNS could support engagement with task-relevant stimuli and facilitate motor learning during PT [40]. However, this hypothesis remains to be tested.

TaVNS has been explored as a potential standalone therapy for PD [41,42,43,44], and has been investigated in combination with PT in other patient populations [28,29,30]. However, taVNS has not previously been tested alongside PT in individuals with PD. Before evaluating efficacy, it is important to first test the safety and feasibility of integrating taVNS with PT treatment. Therefore, this pilot study aimed to establish and test a protocol for combining taVNS with PT in individuals with PD, while exploring preliminary trends in treatment effect to guide future hypothesis testing. This work is intended to inform future trials investigating the therapeutic benefits of this combined intervention.

## 2. Methods

### 2.1. Trial Design and Regulatory Oversight

**Figure 1:**
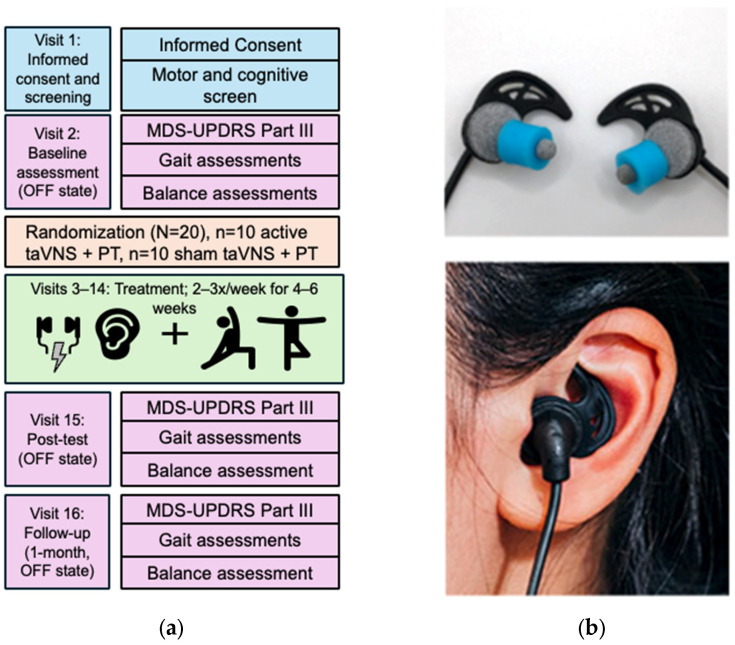
Overview of study design and methods. (**a**) The block diagram illustrates subject flow and treatment through the feasibility evaluation of bilateral transcutaneous auricular vagus nerve stimulation combined with physical therapy for improving Parkinsonian symptoms. (**b**) Hydrogel earbud electrodes used in this study were designed to bilaterally target cranial nerve afferents innervating the skin of the external acoustic meatus.

This study was designed as a pilot, randomized, sham-controlled clinical trial with aims focused on assessing safety, tolerability, and feasibility (Figure 1). Blinding procedures were implemented to evaluate the feasibility of maintaining participant and assessor blinding for a future definitive trial and to reduce the risk of bias in exploratory outcome measures. The study protocol included 16 visits: screening, baseline motor assessment, 12 treatment sessions over six weeks, a post-test assessment, and a four-week follow-up visit. All assessment visits were performed in the OFF-medication state (≥12 h since last dopaminergic medication) to reduce the confounding influence of medication on motor scores. In contrast, all treatment sessions (including taVNS and PT) were completed in the ON medication state to reflect real-world clinical practice and ensure safety during exercise. Participants otherwise continued their usual medical care throughout the study, and no study-related changes to PD medication regimens were required. Cognitive and quality of life outcomes were collected at each assessment but are beyond the scope of this paper. Participants were randomized at a 1:1 allocation ratio into 1 of 2 groups: (1) active taVNS plus PT or (2) sham taVNS plus PT. Written informed consent was obtained prior to enrollment and study participation. The study protocol was approved by the University of Alabama at Birmingham IRB (300010182, approved 8 August 2023) and registered on ClinicalTrials.gov (NCT05871151).

### 2.2. Participants

**Figure 2:**
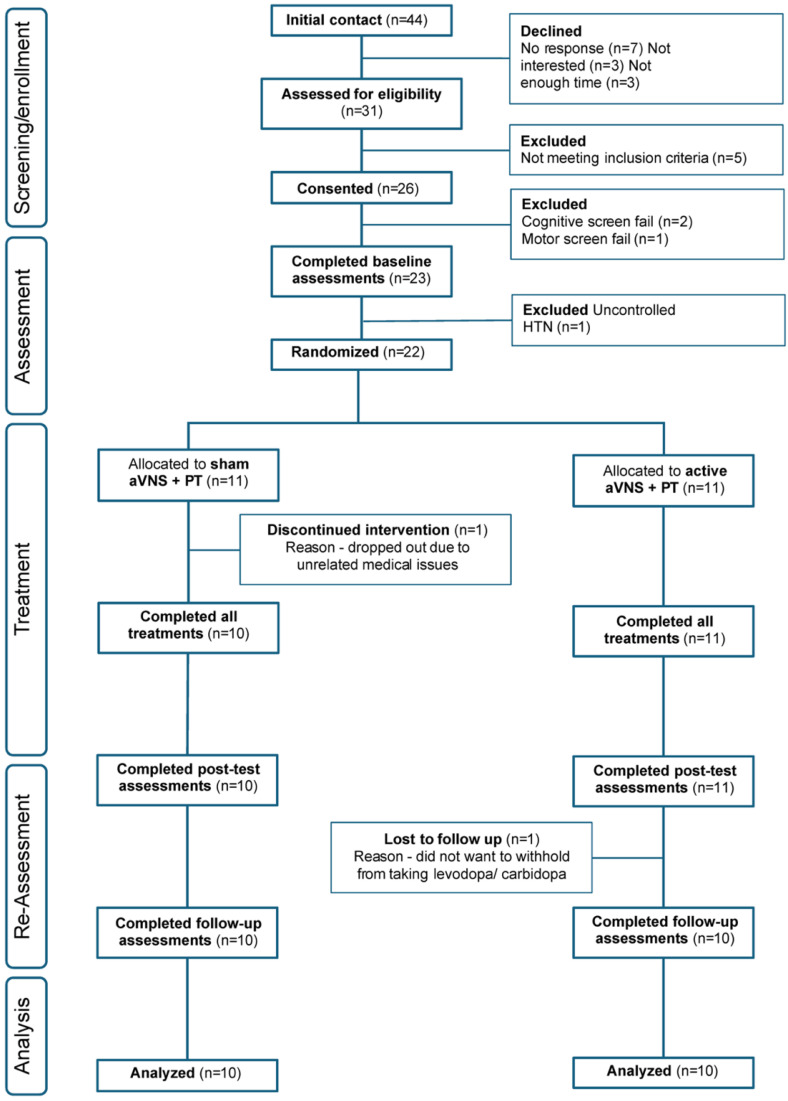
CONSORT flow diagram. The CONSORT flow diagram for the study illustrates participant screening, enrollment, randomization, intervention, and analysis. Of 44 individuals initially contacted, 22 were randomized into either sham or active transcutaneous auricular vagus nerve stimulation (taVNS) treatment groups. A total of 20 participants completed all study procedures, including 12 taVNS plus physical therapy treatment sessions and were included in the final analysis, with reasons for exclusions and attrition documented throughout the flow.

Participants were recruited through UAB’s Movement Disorders Clinic, local support groups, and patient advocacy organizations. Inclusion criteria were (1) neurologist-confirmed diagnosis of idiopathic Parkinson’s disease, (2) age 35–80, (3) stable medications, (4) no falls in 6 weeks, (5) ability to walk without aids, and (6) written approval from the treating neurologist to participate in the study. Exclusion criteria were: (1) moderate-severe cognitive impairment (MoCA < 20), to exclude individuals who may have difficulty providing informed consent and following multi-step instructions; (2) psychiatric comorbidities; (3) clinically significant cardiovascular or neurological comorbidities (e.g., history of myocardial infarction, uncontrolled hypertension, heart failure, significant arrhythmia, stroke, or transient ischemic attack), as determined by chart review and patient history; (4) implanted medical devices; (5) history of seizures and/or vasovagal syncope; and (6) pregnancy. Interested participants first completed a phone eligibility screen. Those eligible were invited for in-person screening and consent. After eligibility was confirmed and physician approval obtained, participants were consented and enrolled (Figure 2).

### 2.3. Interventions

Participants completed 12 treatment sessions (2–3/week) over six weeks, reflecting typical PT care. Each 60 min session began with baseline heart rate and blood pressure (BP) reading, followed by 15 min of active or sham taVNS while resting. Study staff remained present and reassessed HR/BP post-stimulation. Participants then completed a 45 min one-on-one PT session (see below, PWR! Moves PT Treatment) with a PD-trained clinician, followed by post-exercise vitals. The only difference between groups was the electrical current output of the taVNS device.

### 2.4. Transcutaneous Auricular Vagus Nerve Stimulation (taVNS)

Bilateral taVNS was delivered using a small, current-controlled device (vagus.net) connected to conductive, hydrogel earbud electrodes (BRAIN Buds; IST, LLC, Dover, NJ, USA) inserted into the external acoustic meatus of the right and left ears (Figure 1b). Skin was inspected before and after each use. Biphasic pulses (250 µsec pulse durations, 50 µsec inter-pulse interval) were delivered at 30 Hz and 1.0 to 4.0 mA for 15 min. These parameters were selected to align with prior clinical and preclinical taVNS studies in PD and other neurological populations, which have demonstrated acute physiological effects using similar dosing [41,42,44,45,46]. Stimulation duration was selected for clinical feasibility, as 15 min equates to one billable unit of electrotherapy and could more easily be integrated into clinical workflows. Stimulation intensity was individually titrated for comfort at each session, beginning at 4.0 mA. Most participants found this intensity tolerable; however, if discomfort was reported, stimulation intensity was gradually decreased in 0.1–0.5 mA increments until comfort was achieved. Our rationale for this comfort threshold approach was to ensure tolerability across repeated sessions in a pragmatic, clinic-based context. Sham devices mimicked controls but delivered 0 mA current.

### 2.5. PWR! Moves Physical Therapy Treatment

PWR! Moves^®^ (pwr4life.org) is a flexible and adaptable PD-specific exercise program that is increasingly utilized in PT practice. This program was selected as the standard exercise platform for this study to provide a relevant foundation for testing whether adjunctive taVNS can enhance motor outcomes in the clinical setting. Participants in both groups completed 45 min PWR! Moves PT session with a PWR! Moves certified clinician. The program includes 4 basic movements labeled PWR! Up, Rock, Twist, and Step, targeting antigravity extension, weight shifting, axial mobility, and transitional movements. Each movement can be performed in standing, sitting, quadruped, prone, or supine positions. Sessions were tailored to each participant’s ability level, with emphasis on large amplitude and high effort during sessions. Progression and prescription of all movements were determined by the clinician. Patient-specific deficits (whether self-reported or noted during motor function assessments) were considered in treatment sessions.

### 2.6. Outcome Measures

Safety and feasibility metrics included (1) recruitment, enrollment, and attrition rates; (2) adverse events experienced during stimulation or exercise; and (3) treatment tolerability [47]. Adverse events (AEs) were defined as any unintended medical occurrence during or following taVNS or PT. AEs of interest included stimulation-site irritation or discomfort, pain or unpleasant sensory symptoms, autonomic or cardiovascular symptoms such as dizziness, lightheadedness, palpitations, presyncope/syncope, abnormal HR or BP responses. AEs were assessed systematically at each treatment visit through participant self-report, direct observation by study staff, pre- and post-stimulation skin inspection, and measurement of HR and BP before taVNS, immediately after taVNS, and immediately after exercise. Changes in the Movement Disorders Society Unified Parkinson’s disease Rating Scale Motor Sub-Scale (MDS-UPDRS Part III) were assessed over three time points to assess trends in treatment response: (1) baseline (assessment 1), (2) immediately following 12 taVNS + PT sessions (assessment 2), and at 4 weeks follow-up (assessment 3). The MDS-UPDRS Part III was administered at the start of each visit to minimize fatigue effects. Additional outcome measures included a series of clinical tests commonly used in PT practice, including the Modified Clinical Test of Sensory Interaction and Balance (mCTSIB; measure of static balance), Functional Gait Assessment (FGA; measure of dynamic balance, score out of 30, higher scores indicate better balance), Mini Balance Evaluation Systems Test (mini-BEST; measure of static and dynamic balance, score out of 28, higher scores indicate better balance), Six-Minute Walk Test (6MWT; measure of CV endurance, measures distance walked in 6 min in feet), and the 10 m walk test (10MWT-ss and 10MWT-fs, which measures the time taken to walk 10 m at self-selected and fastest gait speeds). Additionally, CV response to stimulation and exercise was measured at each treatment visit by recording HR, systolic blood pressure (SBP), and diastolic blood pressure (DBP) at three time points: (1) pre-taVNS, (2) immediately post-taVNS, and (3) immediately post-exercise. All data was collected on-site at UAB’s Wellness, Health, and Research Facility (WHARF). 

### 2.7. Study Participants

We targeted a sample size of 20 (10 per group) based on the projected number of individuals that could be recruited, screened, enrolled, and retained within the study period. The study PI enrolled participants and assigned participants to interventions using RedCap’s randomization module. Participant blinding was attempted by using a standardized blinding script to minimize participant awareness of group assignment. Due to the small study team and limited personnel resources, the PI was not blinded to group assignment. All other study staff involved in assessments and intervention delivery remained blinded throughout the study to reduce potential bias.

### 2.8. Statistical Analysis

Feasibility and safety outcomes, including recruitment, enrollment, attrition, and adverse events, were summarized using frequency counts and percentages. Descriptive statistics were calculated for baseline demographic and clinical variables. Baseline differences and tolerability were compared using independent *t*-tests and chi-square tests, as appropriate.

This study was not statistically powered to evaluate efficacy; however, we conducted ancillary and exploratory analyses to identify potential trends in motor and CV outcomes to guide protocol refinement and outcome selection for a future, adequately powered clinical trial. For select motor outcomes (MDS-UPDRS Part III, Mini-BESTest, and 6MWT), we utilized linear mixed-effect models to evaluate longitudinal between-group differences. LMMs were constructed with group (active vs. sham), timepoint (baseline, post-treatment, and 4-week follow-up), and the group × timepoint interaction entered as fixed effects. A random intercept for each participant was included in all models to account for within-subject variance across the repeated-measures design. 

To evaluate physiological adaptability, unified 3-timepoint LMMs were employed for CV responses (HR, SBP, and DBP) to map the continuous trajectory across the treatment session. Fixed effects included group, timepoint (pre-taVNS, post-taVNS, and post-exercise), and their interaction, with participant included as a random intercept. All mixed-effects modeling was conducted in R using the lme4 and lmerTest packages, with significance set at *p* < 0.05. Missing data were not imputed; analyses were conducted using available data for each outcome and time point.

To supplement these inferential statistics and clinically interpret the magnitude and retention of intervention effects, descriptive statistics (means, standard deviations, and mean change scores) were also calculated and described. Furthermore, to formally evaluate the clinical significance of between-group differences independent of sample size, standardized effect sizes (Hedge’s g) and 95% confidence intervals (CIs) were calculated for the longitudinal clinical change scores.

## 3. Results

### 3.1. Feasibility

We screened 44 individuals, of whom 33 met preliminary criteria and 26 were consented and enrolled between May 2023 and September 2024. The study was completed once recruitment goals were met. Three subjects were excluded after in-person screening, yielding a 70% enrollment rate (23/33). One participant was excluded pre-randomization due to hypertension. Twenty-two participants were randomized (11 per group). One sham participant withdrew due to an unrelated illness, and one active participant missed follow-up, resulting in a 91% completion rate and a 95% adherence rate. A total of 20 participants (10 per group) were included in the final analyses. Participants in both groups continued their usual care throughout the trial. 

### 3.2. Demographics and Clinical Characteristics

Baseline demographic and clinical characteristics are presented in Table 1. Participants averaged 67.2 ± 7.1 years of age, with PD duration of 5.0 ± 3.2 years and mild-moderate disease severity (H&Y 1–3). PD motor subtype was not formally assessed. There were no significant between-group differences in age (*p* = 0.143, CI: −1.75, 11.15), years since diagnosis (*p* = 0.813, CI: −3.42, 2.72), H&Y stage (*p* = 0.382, CI: −3.42, 2.72), cognitive function (MoCA OFF: *p* = 0.640, CI: −2.71, 1.71; MoCA ON: *p* = 0.723, CI: −2.74, 1.94), and ethnic composition (*p* = 0.305). Sex and gender distributions were identical across groups. There were no significant differences in MDS-UPDRS Part III scores at baseline in the OFF- (sham: 36.7 ± 8.6, active: 31.0 ± 12.68) and ON-medication (sham: 34.2 ± 14.66, active: 26.3 ± 13.21) states (OFF: *p* = 0.255, CI: −4.48, 15.88; ON: *p* = 0.222, CI: −5.21, 21.01). 

### 3.3. Safety and Tolerability of Treatment

Following consent, randomization, and enrollment, participants began the multi-week treatment protocol combining taVNS with PT (Figure 2). AE outcomes were assessed from baseline through the 4-week follow-up visit. There were no AEs reported during the entire duration of the trial. Analysis of tolerability data (Table 2) showed participants in the active group were more likely to perceive stimulation compared to sham (*p* = 0.021). We found 33.3% of participants in the active group reported feeling the stimulation every time and 56.6% most of the time, while the sham group reported only occasional (50.0%) or rare (50.0%) perception of stimulation. Perceived stimulation intensity ratings (0–10 scale) were higher in the active group (3.0 ± 1.89) compared to the sham group (0.9 ± 1.29; *p* = 0.009, CI: −3.62, −0.58). However, the average rating of 3.0 in the active group indicates that the intensity was perceived as relatively mild. Tingling was the most frequently reported sensation (70% active vs. 40% sham), followed by a pulsating sensation (30% active, 0% sham). No participants reported pain or discomfort during any of the treatment sessions. As these results indicate, blinding integrity is difficult to maintain at or above sensory thresholds, and participants in the active group were more likely to correctly identify their stimulation status. 

### 3.4. Effects of taVNS and Exercise on Motor Outcomes

**Figure 3:**
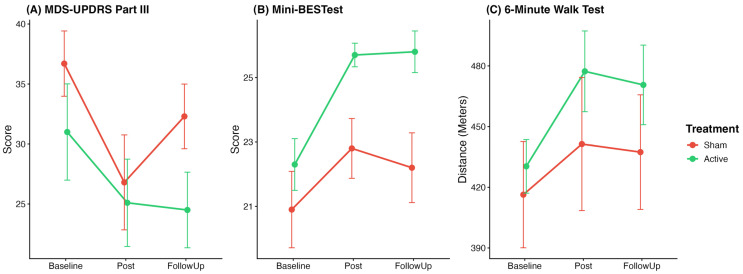
Longitudinal effects of taVNS and physical therapy on motor, balance, and endurance outcomes. (**A**) Movement Disorder Society-sponsored revision of the Unified Parkinson’s Disease Rating Scale (MDS-UPDRS) Part III motor scores. (**B**) Mini-Balance Evaluation Systems Test (Mini-BESTest) scores. (**C**) 6-min Walk Test (6MWT) distance, reported in meters. Data are presented as mean ± standard error (SE) at baseline (BL), immediately post-treatment (post), and at the 4-week follow-up (follow-up). Statistical evaluation utilized linear mixed-effects models (LMMs) controlling for baseline age. A highly significant main effect of Time was observed across all three metrics (*p* < 0.05), reflecting overall improvements following the physical therapy intervention in both groups.

To characterize preliminary treatment-related trends, we examined changes in select motor (MDS-UPDRS Part III), balance (mini-BEST), and walking capacity (6MWT) outcomes across time in the active and sham taVNS groups (Figure 3). For the primary motor outcome, the MDS-UPDRS Part III, the LMM revealed a highly significant main effect of Time (F(2,36) = 9.54, *p* = 0.0004), indicating overall motor improvement in both treatment groups. The group × timepoint interaction did not reach statistical significance (F(2,36) = 1.40, *p* = 0.259); however, descriptive analysis of the clinical trajectories provides additional insight. Immediately following the intervention (post-treatment), both groups achieved a clinically meaningful reduction in motor symptom severity (defined as a decrease of ≥5 points [48]. The sham group improved by an average of 9.9 points (95% CI: −16.1, −3.7; baseline: 36.7 ± 8.6; post: 26.8 ± 12.5), while the active taVNS group improved by 5.9 points (95% CI: −11.6, −0.2; baseline: 31.0 ± 12.7; post: 25.1 ± 11.5). However, at the 4-week follow-up, the sham group experienced a clinical regression in motor function, losing 5.5 points of their initial post-treatment improvement (95% CI: −2.7, 13.7; follow-up: 32.3 ± 8.5). In contrast, the active taVNS group maintained their therapeutic gains, exhibiting minimal loss of motor function between the post-treatment and follow-up assessments (follow-up: 24.5 ± 10.0; 95% CI: −4.8, 3.6). This maintenance of motor function in the active cohort yielded a moderate-to-large between-group effect size during the retention phase (Hedge’s g = 0.64).

Analysis of balance via the Mini-BESTest similarly demonstrated a highly significant main effect of Time (F(2,36) = 12.73, *p* < 0.001). However, again, the overarching group × timepoint interaction was not statistically significant (F(2,36) = 1.87, *p* = 0.167). Descriptive analysis of the clinical change scores indicates that following treatment, the active taVNS group demonstrated an average improvement of 3.4 points (95% CI: 1.6, 5.2; baseline: 22.3 ± 2.5; post: 25.7 ± 1.2), achieving the Minimal Clinically Important Difference (MCID) of 3 to 4 points for the Mini-BESTest in PD [49]. In contrast, the sham group exhibited a sub-clinical initial improvement of only 1.9 points (95% CI: −0.7, 4.5; baseline: 20.9 ± 3.8; post: 22.8 ± 2.9, Hedge’s g = 0.47). During the 4-week retention phase, both groups remained relatively stable, with the sham group losing 0.6 points of their initial gains (95% CI: −1.7, 0.5; follow-up: 22.2 ± 3.4) and the active group maintaining their gains (gaining 0.1 points, 95% CI: −1.0, 1.2; follow-up: 25.8 ± 2.0). This slight divergence in stability represented a moderate retention effect size (Hedge’s g = 0.43).

Finally, analysis of walking capacity measured by the 6MWT revealed a significant main effect of Time (F(2,33.3) = 7.05, *p* = 0.002), reflecting overall improvements across both groups. Consistent with the other motor metrics, there was no significant group × timepoint interaction (F(2,33.3) = 1.01, *p* = 0.371). However, descriptive analysis of the clinical change scores reveals a clinically important difference in the initial therapeutic response of the active group only. Following the intervention, the active taVNS group improved their 6MWT distance by a mean of 53.6 m (95% CI: 16.3, 90.9; baseline: 430.4 ± 42.0 m; post: 477.3 ± 59.9 m), effectively achieving the MCID threshold of 54 m for PD patients. In contrast, the sham group exhibited a sub-clinical initial improvement of only 19.7 m (95% CI: −18.5, 57.9; baseline: 416.4 ± 83.1 m; post: 441.4 ± 98.7 m). This indicates that the active group achieved a reliable and robust initial response, yielding a moderate-to-large between-group effect size (Hedge’s g = 0.66). During the 4-week follow-up period, both groups experienced a regression in functional walking capacity (active group: −18.5 m, 95% CI: −71.0, 34.0; sham group: −5.9 m, 95% CI: −35.4, 23.6; Hedge’s g = 0.22). 

Collectively, these longitudinal analyses confirm that structured physical therapy significantly improves motor, balance, and endurance metrics in PD. Additionally, while inferential statistics did not definitively separate the groups in this small pilot, the moderate-to-large effect sizes and reliable confidence intervals observed in the active cohort suggest that pairing PT with active taVNS may amplify the initial magnitude of clinical response towards clinically meaningful change, and improve the long-term retention of therapeutic benefits compared to sham stimulation. However, these findings remain speculative and must be confirmed with larger clinical trials.

### 3.5. Effects of taVNS and Exercise on Cardiovascular Outcomes

**Figure 4:**
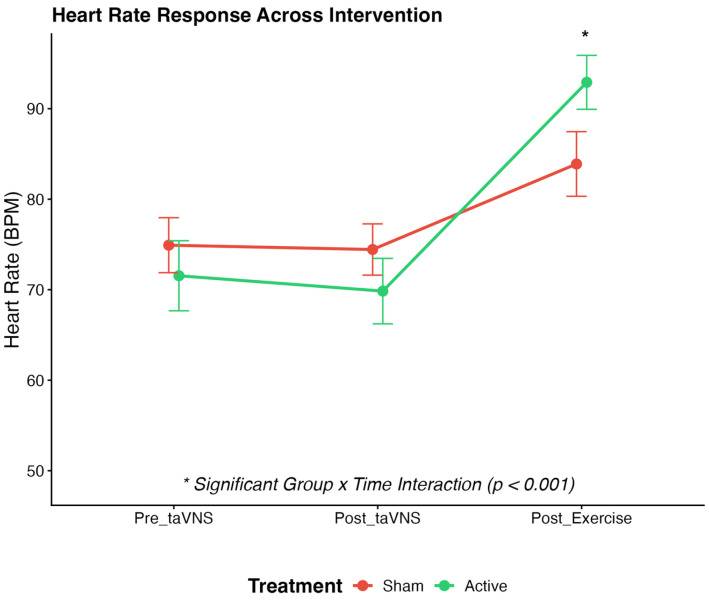
Heart rate response across the combined taVNS and physical therapy intervention. Data points represent the mean ± standard error (SE) heart rate in beats per minute (bpm) for the active taVNS (green) and sham (red) groups. Measurements were recorded at baseline (pre-taVNS), immediately following 15 min of resting stimulation (post-taVNS), and immediately following 45 min of targeted physical therapy (post-exercise). A unified 3-timepoint linear mixed-effects model revealed a highly significant group × timepoint interaction (*p* < 0.001, denoted by *). During the resting intervention period, both groups exhibited a uniform, non-significant decrease in heart rate. However, during the subsequent physical therapy session, the active taVNS group demonstrated a significantly augmented chronotropic response to physical exertion, increasing by an average of ~23.1 bpm compared to the sham group’s ~9.5 bpm increase.

To explore the effect of taVNS on cardiovascular physiological responses at rest and during exercise, average HR, SBP, and DBP were recorded prior to taVNS (pre-taVNS), after 15 min of active or sham stimulation at rest (post-taVNS), and following 45 min of PT (post-exercise) across all 12 visits (Figure 4). LMMs were used to explore differences between groups, and descriptive statistics, along with 95% CIs and effect size, were used to describe trends in the data (Table 3). Baseline (pre-taVNS) vitals reflected the participants’ average resting states, with the LMMs confirming no significant baseline differences between the active and sham groups in HR (*p* = 0.483), SBP (*p* = 0.691), or DBP (*p* = 0.630). 

Analysis of HR data revealed a highly significant group × timepoint interaction (F(2,36) = 20.40, *p* = 1.19 × 10^−6^). Exploration of fixed effects revealed that both groups exhibited a slight, non-significant dip in HR immediately after both active and sham taVNS compared to baseline, with no significant difference between groups (*p* = 0.608). During this acute resting phase, the sham group’s HR decreased, on average, by 0.5 bpm (95% CI: −1.6, 0.6). In contrast, the active group’s HR decreased by 1.7 bpm (95% CI: −3.2, −0.2; Hedge’s g = 0.64). However, following PT (post-exercise), the active taVNS group demonstrated a significantly more robust HR response to exercise compared to sham (*p* = 6.88 × 10^−6^). The sham group’s HR increased by an average of 9.5 bpm (95% CI: 5.7, 13.3) immediately after physical exertion, whereas the active taVNS group’s HR increased by 23.1 bpm (95% CI: 18.4, 27.8). This distinct divergence in physiological reactivity represents a large between-group effect size (Hedge’s g = 2.18).

Conversely, the LMMs for both SBP and DBP revealed highly significant main effects of Time (SBP: F(2,36) = 25.00, *p* < 0.001; DBP: F(2,36) = 11.44, *p* < 0.001), but did not demonstrate a significant group × timepoint interaction (SBP: *p* = 0.774; DBP: *p* = 0.467). During the acute resting stimulation, the sham group’s SBP decreased on average by 7.7 mmHg (95% CI: −13.2, −2.2), whereas the active group’s SBP decreased by 8.7 mmHg (95% CI: −12.3, −5.1; Hedge’s g = 0.15). DBP pre and post stimulation followed similar trends, with the sham group demonstrating a 1.3 mmHg decrease (95% CI: −3.7, 1.1) and the active group demonstrating a 2.8 mmHg decrease (95% CI: −4.4, −1.2; Hedge’s g = 0.51). After exercise, the sham group’s SBP increased by 13.2 mmHg (95% CI: 7.2, 19.2), whereas the active group’s SBP increased by 16.1 mmHg (95% CI: 6.4, 25.8; Hedge’s g = 0.25). Finally, DBP after exercise increased by 2.6 mmHg in the sham group (95% CI: 0.1, 5.1), and 4.2 mmHg in the active group (95% CI: 1.3, 7.1; Hedge’s g = 0.39). Collectively, these findings indicate that active taVNS may function as an autonomic primer, engaging resting parasympathetic tone while amplifying the necessary sympathetic HR response to physical exertion, without causing concurrent, unsafe exaggerations in blood pressure.

## 4. Discussion

This pilot randomized, sham-controlled trial primarily evaluated the safety, tolerability, and feasibility of combining taVNS with PT in individuals with PD. Electrical stimulation modalities have been used alongside therapeutic exercise in rehabilitation practice for decades [50]. Although taVNS targets different neural pathways than conventional stimulation approaches, its use of familiar electrotherapeutic principles may facilitate a more rapid integration into rehabilitation workflows, helping to bridge the 17-year gap between research and clinical practice [51]. Combining VNS with PT is of increasing interest, particularly given the promising findings from paired invasive VNS + rehab studies in stroke [31,33,52,53,54,55] and spinal cord injury [52,56,57]. Additionally, the impact of taVNS on motor and non-motor PD symptoms has been explored [41], with recent studies demonstrating acute and significant effects on gait and balance [42,43,44,58]. However, the use of taVNS in combination with PT remains a relatively new area of study, and additional work is needed to determine how this therapeutic approach can be implemented to synergistically improve patient outcomes. Therefore, the present study aims to systematically evaluate whether taVNS can be delivered repeatedly within a conventional PT workflow for individuals with PD.

To our knowledge, this is the first study to evaluate the safety, tolerability, and feasibility of combining taVNS with PT in individuals with PD. The primary finding was that the combined intervention was safe, well-tolerated, and feasible within a clinical rehabilitation context. Given the high prevalence of dysautonomia in PD [59], and because taVNS can influence ANS function [60,61,62,63,64,65], evaluating the safety of administering taVNS in close temporal proximity to exercise was particularly important. Our goal was to ensure that taVNS did not produce adverse autonomic effects, such as orthostatic hypotension, during or after treatment. No adverse events occurred throughout the study period, and no participants required discontinuation of stimulation or exercise because of autonomic symptoms, pain, or discomfort. Additionally, administering taVNS prior to PT did not interfere with treatment delivery or prevent participants from engaging in the prescribed PT sessions. These findings provide preliminary support for advancing this combined intervention to the next stage of testing in individuals with PD. However, because this was a small pilot study that excluded individuals with significant cardiovascular comorbidities and more severe or atypical PD presentations, these findings should be interpreted as preliminary evidence of short-term safety in a screened mild-to-moderate PD sample rather than definitive evidence of safety across the broader PD population. 

Participant tolerability was also an important consideration. The long-term goal of this work is to evaluate the integration of taVNS into routine clinical practice. Patient tolerability is a necessary first step toward clinical translation, as even highly promising interventions are unlikely to be adopted if they are uncomfortable, burdensome, or disruptive to care. This study used a proprietary hydrogel earbud electrode designed to improve participant comfort; however, the tolerability of this electrode configuration in individuals with PD had not been established. No participants reported pain or discomfort, and perceived stimulation intensity in the active group was mild on average. Importantly, tolerability was maintained across a repeated, multi-week treatment protocol rather than a single laboratory exposure, supporting the viability of this approach for future rehabilitation studies.

However, the tolerability findings also highlight an important feasibility challenge for future efficacy trials. Participants in the active group were more likely to perceive stimulation and reported higher stimulation intensity than participants in the sham group, indicating that blinding integrity may be difficult to maintain when stimulation is delivered at or above sensory threshold. Although the selected stimulation intensity did not appear to compromise tolerability, it may introduce expectancy effects in future trials designed to evaluate efficacy. Future studies should therefore consider protocol refinements such as sub-sensory stimulation, improved active sham approaches, or formal measurement of treatment expectancy and blinding integrity. These refinements will be important for distinguishing the specific effects of taVNS from nonspecific effects related to stimulation perception, attention from study staff, or participant expectations.

Feasibility was another central objective of this pilot study. Specifically, we sought to determine whether participants could be recruited, enrolled, retained, and treated within a multi-week protocol combining taVNS with PT, given the safety considerations and eligibility criteria relevant to VNS research. Overall, the recruitment, retention, and adherence outcomes suggest that this intervention model is feasible within a rehabilitation research setting. Eligible participants were willing to enroll in a repeated-session neuromodulation + PT protocol, and most participants completed the study with high adherence. These findings suggest that taVNS can be integrated into a multi-week PT intervention without imposing excessive participant burden or disrupting treatment delivery. 

Although the 15 min taVNS protocol was feasible and well tolerated, future studies should examine whether this stimulation duration is sufficient to maximize rehabilitation-related effects. The optimal dose-duration for taVNS remains unknown, and many studies demonstrating acute autonomic or neurophysiologic effects have used stimulation periods of approximately 30 min or longer [66]. Therefore, the 15 min duration used in this pragmatic protocol may have prioritized clinical feasibility at the expense of physiologic effect size. Future studies should directly compare stimulation durations to determine whether longer pre-session dosing produces stronger or more durable rehabilitation-related effects.

Future studies should also consider whether taVNS should be delivered before PT as a priming strategy or paired directly with the PT intervention. Pairing stimulation with movement is the approach used in the majority of invasive VNS rehabilitation studies [30,31,52,67], as evidence suggests that the timing of stimulation relative to successful movement execution may be critical for optimizing therapeutic effects [68]. In the present study, we selected a pre-session priming approach because it was most compatible with the device, treatment workflow, and feasibility aims of this pilot trial. However, this approach may not produce the same temporally specific reinforcement of task-relevant motor circuits proposed in paired VNS models. Future studies may consider developing systems that can identify successful movement repetitions or clinically meaningful movement patterns and deliver stimulation precisely in relation to those events.

Although this pilot study was not powered to establish efficacy, the exploratory motor findings revealed clinically relevant patterns that warrant further investigation. Across outcomes, structured PT was associated with improvements in motor function, which is expected given that all participants received an active rehabilitation intervention. However, the trajectory of change differed between groups. For motor symptom severity, measured by the MDS-UPDRS Part III, both groups improved immediately following treatment, but only the active taVNS group maintained these gains at the 4-week follow-up. A similar pattern was observed for balance performance. Although Mini-BESTest scores improved in both groups, the magnitude of change was greater in the active group vs. sham. Only the active taVNS group achieved a clinically meaningful change. Additionally, these gains were maintained at follow-up in the active group, whereas the sham group demonstrated less durable improvement. This finding is particularly relevant in PD, where balance impairment and falls are common contributors to reduced mobility, injury risk, loss of independence, and diminished quality of life [69]. Consequently, interventions that improve balance may therefore have meaningful clinical implications. 

The retention of improvements in motor function and balance reflects a synergistic interaction between the LC–norepinephrine (LC-NE)-mediated neuroplastic effects of taVNS [28,29,30,34,70] and the task-specific neuroplasticity induced by PWR! Moves, which emphasizes large-amplitude, dynamic balance training. One plausible mechanism to support these results is that taVNS “primes” the brain by activating the LC-NE system prior to therapy, increasing arousal, facilitating synaptic plasticity, and enhancing consolidation of the motor skills practiced during PT. This concept parallels findings from stroke rehabilitation research, where VNS paired with task-specific training produced more robust and durable motor gains than training alone [31]. However, it is also possible that optimal effects require “pairing” stimulation with successful movement execution, timing NE release to coincide with relevant neural activity. Determining whether priming, pairing, or a combination yields the most potent neuroplastic response represents an important avenue for future research.

Finally, submaximal aerobic/functional walking capacity, measured by the 6MWT, improved in both groups, but only the active taVNS group achieved a clinically meaningful change immediately following treatment. This pattern may suggest a greater short-term cardiovascular benefit of the combined intervention. However, unlike the motor severity and balance outcomes, improvements in the 6MWT were not maintained at follow-up in either group. This may indicate that endurance-related benefits require continued intervention or more targeted cardiovascular training to be sustained over time. Overall, these exploratory findings suggest that taVNS combined with PT may have the potential to enhance the magnitude and/or retention of rehabilitation-related gains, but larger, adequately powered trials are needed to determine whether these patterns reflect true treatment effects.

The greater HR response to exercise observed in the active taVNS group is of particular interest, given the high prevalence of cardiovascular dysautonomia in PD [59]. Individuals with PD commonly demonstrate blunted or abnormal HR responses to exercise as a result of degeneration of neural structures of the ANS [71,72], which can complicate exercise prescription and limit the ability to achieve target exercise intensities. In this context, taVNS delivered immediately before PT may have acutely modulated autonomic function in a way that supported a more robust CV response during exercise. Importantly, this larger HR response was not accompanied by symptoms suggestive of cardiovascular intolerance. Participants in the active group did not report palpitations, shortness of breath, dizziness, or lightheadedness, suggesting that the observed HR increase occurred within a clinically tolerable range.

The mechanism underlying this response remains unclear. Because taVNS is often discussed in relation to parasympathetic modulation, one might expect stimulation to reduce HR at rest, as was observed immediately following taVNS. However, the larger HR response during subsequent exercise suggests that taVNS may not simply exert a unidirectional parasympathetic effect. Instead, taVNS may have altered the autonomic state in a way that improved cardiovascular adaptability, allowing participants to transition more effectively from rest to exercise. This could reflect enhanced parasympathetic withdrawal, greater sympathetic engagement during exertion, improved baroreflex responsiveness, or a combination of these mechanisms. These explanations remain speculative and should be tested in future studies using continuous HR monitoring, HRV, direct measures of exercise intensity, and more comprehensive autonomic assessment. Overall, the larger but clinically tolerable HR response observed in the active group supports further investigation of taVNS as a potential strategy for improving cardiovascular responsiveness during rehabilitation in PD.

Several limitations should be considered when interpreting the findings of this pilot trial. First, although the study demonstrated that taVNS could be feasibly integrated into a PT workflow, several feasibility-related limitations remain. The study PI was involved in multiple aspects of the trial, including recruitment, enrollment, assessment, and treatment delivery. This was due to the small study team and limited financial resources; however, the lack of PI blinding may have introduced bias, particularly for clinician-rated and performance-based outcomes. Future studies should incorporate blinded assessors and separate personnel for stimulation delivery, intervention administration, and outcome assessment. Additionally, blinding integrity was difficult to maintain, likely due to the use of supra-sensory stimulation thresholds. Participants in the active group were more likely to perceive stimulation, which may have introduced expectancy effects. Future studies should consider sub-sensory or sub-perceptual stimulation intensities, improved active sham approaches, and formal assessment of treatment expectancy and blinding integrity.

Second, the exploratory motor outcomes should be interpreted cautiously. Participants were not instructed to restrict or standardize exercise or physical activity outside of the treatment sessions or during the follow-up period. Therefore, we cannot determine whether changes in MDS-UPDRS Part III, Mini-BESTest, or 6MWT performance were influenced by differences in participants’ exercise behavior outside of the study intervention. This is particularly relevant for follow-up outcomes, as continued exercise after the treatment period may have contributed to retention of gains in some participants, whereas reduced activity may have contributed to regression in others. Additionally, although the individualized PT intervention improved ecological validity, it introduced variability in exercise intensity, task content, number of repetitions, progression, and motor learning dose across participants. Future studies should incorporate objective physical activity monitoring, structured exercise logs, standardized documentation of treatment intensity and progression, and treatment fidelity procedures to better characterize total exercise exposure.

Third, the cardiovascular findings were limited by the timing and scope of physiological measurement. HR, SBP, and DBP were measured only at discrete time points rather than continuously monitored, limiting our ability to characterize dynamic autonomic responses during stimulation, exercise, and recovery. The PT intervention was also not a standardized aerobic exercise protocol, and individualized target HR zones were not prescribed. Therefore, HR responses to exercise were not standardized across participants. Future studies should examine the effects of taVNS during standardized aerobic exercise protocols and incorporate continuous HRV, beat-to-beat BP, perceived exertion, fatigue, exercise intensity, and cardiorespiratory fitness outcomes to determine whether taVNS meaningfully improves autonomic regulation during rehabilitation.

Finally, although the rationale for pre-session taVNS included potential effects on attention, arousal regulation, autonomic function, and motor learning, these mechanisms were not directly assessed in the present study. Specifically, this study did not include measures of HRV, baroreflex sensitivity, perceived stress, attention, cognitive-motor engagement, LC–NE activity, or neuroplasticity. Therefore, the observed cardiovascular and motor trends should be interpreted as preliminary clinical signals rather than mechanistic evidence. Future studies should include mechanistic outcomes to determine whether taVNS enhances rehabilitation by improving attention to motor practice, reducing maladaptive arousal, improving autonomic flexibility, or some combination of these pathways.

Overall, this study established the preliminary safety, tolerability, and feasibility of combining taVNS with PT in individuals with PD. This study provides necessary early evidence that repeated taVNS can be delivered safely and acceptably alongside PT in a screened PD sample. These findings justify future mechanistic and adequately powered trials designed to determine optimal stimulation dose, timing, sham control, and the extent to which taVNS meaningfully augments rehabilitation outcomes. If effective, this therapeutic approach could be integrated into clinical practice as a scalable adjunct to improve the therapeutic impact of PT and help individuals with PD derive greater benefit from rehabilitation. 

## 5. Conclusions

This pilot randomized, sham-controlled trial provides preliminary evidence that taVNS can be safely and feasibly integrated with PT for individuals with PD. The intervention was well tolerated, with no adverse events attributable to stimulation or exercise. Although not powered to determine efficacy, exploratory findings suggest that taVNS may enhance the therapeutic benefits of PT, and may increase HR responsiveness during exercise without destabilizing SBP or DBP. Future studies should optimize stimulation dosing, strengthen blinding, incorporate continuous cardiovascular monitoring, and evaluate taVNS with standardized aerobic exercise protocols. If effective, taVNS could represent a scalable adjunct to enhance PT outcomes in individuals with PD.

## Figures and Tables

**Table 1:** Demographics and clinical characteristics by treatment group.

Characteristic	Total (N = 20)	Sham (***n*** = 10)	Active (***n*** = 10)	***p***-Value	95% CI
Age, years	67.2 (7.10)	69.5 (6.15)	64.8 (7.51)	0.143	−1.75, 11.15
Years since diagnosis	5.0 (3.18)	4.8 (3.05)	5.2 (3.47)	0.813	−3.42, 2.72
Hoehn and Yahr stage	2.7 (0.75)	2.8 (0.63)	2.5 (0.85)	0.382	−0.40, 1.00
MDS-UPDRS Part III (off)	33.9 (10.95)	36.7 (8.6)	31.0 (12.68)	0.255	−4.48, 15.88
MDS-UPDRS Part III (on)	30.3 (14.17)	34.2 (14.66)	26.3 (13.21)	0.222	−5.21, 21.01
MoCA (off)	27.0 (2.31)	26.7 (1.95)	27.2 (2.70)	0.640	−2.71, 1.71
MoCA (on)	26.7 (2.43)	26.5 (2.37)	26.9 (2.60)	0.723	−2.74, 1.94
Sex					
Male	16 (80.0%)	8 (80.0%)	8 (80.0%)	1.000	
Female	4 (20.0%)	2 (20.0%)	2 (20.0%)		
Ethnicity					
White/Caucasian	19 (95.0%)	10 (100.0%)	9 (90.0%)	0.305	
Black/African American	1 (5.0%)	0 (0.0%)	1 (10.0%)		

**Table 2:** Tolerability of bilateral transcutaneous auricular vagus nerve stimulation. * indicates *p*-value <0.05.

	Sham (***n*** = 10)	Active (***n*** = 10)	***p***-Value
Feels Stimulation			0.021 *
Yes	0 (0.0%)	4 (40.0%)	
Sometimes	4 (40.0%)	4 (50.0%)	
No	6 (60.0%)	1 (10.0%)	
If yes/sometimes, frequency felt			0.020 *
Every time	0 (0.0%)	3 (33.3%)	
Most of the time	0 (0.0%)	5 (55.6%)	
Occasionally	2 (50.0%)	1 (11.1%)	
Rarely	2 (50.0%)	0 (0.0%)	
Stimulation Intensity (0–10)	0.9 (1.29)	3.0 (1.89)	0.009 *
Tingling Sensation			0.178
Yes	4 (40.0%)	7 (70.0%)	
No	6 (60.0%)	3 (30.0%)	
Pulsating Sensation			0.060
Yes	0 (0.0%)	3 (30.0%)	
No	10 (0.0%)	7 (70.0%)	
Warm Sensation			1.000
Yes	0 (0.0%)	0 (0.0%)	
No	10 (100.0%)	10 (100.0%)	
Pressure			1.000
Yes	0 (0.0%)	0 (0.0%)	
No	10 (100.0%)	10 (100.0%)	
Pain			1.000
Yes	0 (0.0%)	0 (0.0%)	
No	10 (100.0%)	10 (100.0%)	
Dull Ache			1.000
Yes	0 (0.0%)	0 (0.0%)	
No	10 (100.0%)	10 (100.0%)	
Numbness			1.000
Yes	0 (0.0%)	0 (0.0%)	
No	10 (100.0%)	10 (100.0%)	
Overall Tolerability of Treatment			0.060
Very comfortable	8 (80.0%)	6 (60.0%)	
Comfortable	0 (0.0%)	3 (30.0%)	
Neutral	2 (20.0%)	1 (10.0%)	
Uncomfortable	0 (0.0%)	0 (0.0%)	
Very uncomfortable	0 (0.0%)	0 (0.0%)	

**Table 3:** Cardiovascular adaptability and physiological reactivity during the physical therapy intervention. All values are reported as mean ± standard deviation.

Measure	Group	Pre-taVNS (Mean ± SD)	Post-taVNS (Mean ± SD)	Post-Exercise (Mean ± SD)	Exercise Delta (Δ)
Heart rate	Sham	71.5 ± 12.2	69.8 ± 11.4	92.9 ± 9.4	+23.1
(bpm)	Active	74.9 ± 9.6	74.4 ± 9.0	83.9 ± 11.3	+9.5
Systolic BP	Sham	130.2 ± 9.9	121.5 ± 6.9	137.6 ± 15.1	+16.1
(mmHg)	Active	128.5 ± 12.5	120.8 ± 11.8	134.0 ± 14.9	+13.2
Diastolic BP	Sham	75.9 ± 6.1	73.1 ± 5.0	77.3 ± 6.7	+4.2
(mmHg)	Active	76.8 ± 6.2	75.4 ± 6.5	78.1 ± 7.2	+2.6

Values represent the mean ± standard deviation for heart rate (HR), systolic blood pressure (SBP), and diastolic blood pressure (DBP) at baseline, following 15 min of seated stimulation, and immediately following the physical therapy exercise. The Exercise Delta (Δ) column denotes the absolute change from the resting post-taVNS state to the post-exercise state, illustrating the magnitude of physiological reactivity to the physical challenge.

## Data Availability

Dataset available on request from the authors.
